# Effects of Physical Exercise on Executive Functions among College Students in China: Exploring the Influence of Exercise Intensity and Duration

**DOI:** 10.3390/bs13120987

**Published:** 2023-11-29

**Authors:** Ming Yu, Xinyi Han, Xiaomei Wang, Rongxin Guan

**Affiliations:** 1 Physical Education Department, Northeastern University, Shenyang 110819, China; yuming@pe.neu.edu.cn (M.Y.); wangxiaomei@pe.neu.edu.cn (X.W.); 2 College of Physical Education, Hebei Normal University, Shijiazhuang 050024, China; hanxinyi@hebtu.edu.cn

**Keywords:** college students, exercise intensity, exercise duration, executive functions

## Abstract

Background: This study investigates the effects of exercise intensity and duration on executive functions among college students in China. Method: Participants in this study were Chinese college students divided into four groups based on exercise duration and intensity. Each group engaged in physical exercise twice a week for six weeks. Group 1 performed low-intensity exercises for 10 min per session; Group 2 performed low-intensity exercises for 20 min per session; Group 3 performed high-intensity exercises for 10 min per session; and Group 4 performed high-intensity exercise for 20 min per session. Executive functions were assessed in all subjects before the experiment (time 1), after one exercise session (time 2), at the end of the exercise program (time 3), and six weeks after the exercise program (time 4). A mixed ANOVA with a 2 (exercise intensity: low/high) × 2 (duration: short/long) × 4 (time of measurement: time 1/time 2/time 3/time 4) design was employed, with exercise intensity and exercise duration as independent variables and executive functions as the dependent variable. Results: Mixed ANOVA showed that the results revealed a significant main effect of measurement time on working memory accuracy (*p* < 0.001) and reaction time (*p* < 0.001); inhibition control accuracy (*p* < 0.001) and reaction time (*p* < 0.001); cognitive flexibility accuracy (*p* < 0.001) and reaction time (*p* < 0.001). A single session of high-intensity exercise significantly improved executive functions in college students. Both low-intensity and high-intensity exercise were effective in enhancing executive functions, with high-intensity exercise demonstrating better maintenance of the effect. Conclusion: Both exercise intensity and exercise duration were found to enhance executive functions in college students, with exercise intensity showing greater effectiveness than exercise duration.

## 1. Introduction

Executive functions refer to cognitive processes that involve conscious control over thoughts and actions [1]. Executive functions are crucial for problem-solving in daily life. Thus, executive functions significantly impact an individual’s mental health. Impairments in executive functions can result in cognitive, affective, and social dysfunctions. Zelazo, P.D. studies have demonstrated the malleability of executive functions, which increase with age and decline in old age [2]. Consequently, enhancing executive functions is a current research focus. Executive functions encompass three primary components: working memory, inhibitory control, and cognitive flexibility. Working memory serves as a limited-capacity system for the temporary processing and storage of information, playing a crucial role in various complex cognitive activities [3]. Inhibitory control refers to an individual’s capacity to disregard irrelevant stimuli while pursuing cognitive representational goals [4]. Cognitive flexibility entails the ability of individuals to adapt their thinking flexibly to meet the demands presented by new problems or situations [5]. Executive function deficits manifest as difficulties in planning, creative problem-solving, rule adherence, and multitasking. Impairments in executive function are frequently observed in various neurodegenerative disorders, including vascular dementia, Alzheimer’s disease, Parkinson’s disease dementia, progressive supranuclear palsy, Lewy body dementia, and frontotemporal lobe dementia [6].

Physical exercise has been shown to enhance executive functions in individuals. Kong et al. demonstrated that sprint interval exercise enhances executive function, while severe hypoxic exercise does not impair it [7]. Shirzad et al. observed that exercise increases cerebral blood flow, and increased cerebral blood flow may be able to improve an individual’s executive functions [8]. Lebeau et al. demonstrated that acute exercise improved executive functions in American older adults [9]. A randomized controlled trial indicated that 12 months of high- and moderate-intensity exercise did not enhance executive functions in older adults but significantly improved cardiorespiratory fitness [10]. These findings indicate conflicting results regarding the impact of exercise on executive functions in older adults. Huang et al. conducted a systematic review study and found that moderate-intensity acute resistance exercise improved executive functions more effectively than mild and vigorous-intensity exercise [11]. A meta-analysis revealed that aerobic physical exercise significantly enhanced working memory, cognitive flexibility, and inhibitory control in older adults. Moreover, longer durations of aerobic exercise were associated with greater improvements in executive functions [12]. However, another meta-analysis reported that the effect of physical activity on executive functions is modest and varies across different subfunctions [13]. Therefore, based on the aforementioned analysis, there is an urgent need for high-quality research to examine the effects of physical exercise on executive functions.

Despite some controversy surrounding the effects of physical exercise on enhancing executive functions in individuals, the majority of current research supports the notion that physical exercise indeed improves executive functions. Two studies have demonstrated that exercise intensity, a crucial component of physical exercise, significantly influences individuals’ affective and executive functions [14,15]. Zhu et al. demonstrated that high-intensity interval training yielded superior improvements in executive functions compared to moderate-intensity continuous training. Notably, measurements taken 10 min after exercise showed improved executive functions, while immediate post-exercise measurements did not reflect such improvements [16]. Albuquerque et al. investigated the association between acute exercise and executive functions, revealing the beneficial impact of exercise intensity on executive functions [17]. Slusher et al. utilized the Wisconsin card sorting task to assess executive functions in healthy male college students, revealing the significant enhancement of executive functions through high-intensity interval training [18]. Wang et al. employed varied doses of high-intensity interval training as interventions for college students, demonstrating that a moderate dose of such training yielded superior improvements in executive functions [19]. Tsukamoto et al. revealed that, compared to low-intensity physical exercise, moderate-intensity physical exercise is more effective in enhancing executive functions in healthy males. Moreover, it was found that moderate-intensity exercise led to sustained improvements in executive functions during post-exercise recovery [20]. The aforementioned studies have consistently shown that higher exercise intensity corresponds to greater improvements in individual executive function. However, contrasting studies also exist. Costello et al. found that high-intensity interval training impairs executive functions and raises the likelihood of sports injuries in rugby players [21]. Moeller et al. reported that a 20 min session of moderate-intensity exercise positively influenced subjects’ inhibitory control, whereas high-intensity exercise had no impact on inhibitory control [22]. Hence, the influence of exercise intensity on enhancing individual executive functions remains an area that requires further investigation.

Several studies have indicated that exercise duration has an impact on an individual’s executive functions. Chen et al. demonstrated that acute moderate-intensity aerobic exercise yielded superior improvements in individuals’ executive functions compared to exercise sessions lasting 10–45 min [23]. Similarly, Chang et al. arrived at the same conclusion as Chen, highlighting that a 20 min session of physical exercise yields superior improvements in individuals’ executive functions [24]. Another study by Chang et al. demonstrated that there was no significant difference in the improvement of subjects’ executive functions between exercise durations of 30 and 40 min, suggesting comparable effects [25].

The aforementioned research highlights the necessity of conducting comprehensive studies on the effects of physical exercise on college students’ executive functions. Furthermore, further investigation is required to explore the impact of exercise intensity and duration on individual executive functions. To investigate the effects of exercise intensity and duration on college students’ executive functions, this study incorporated varying exercise intensity and duration as independent variables. The choice of college students as participants in this study was based on several factors. Firstly, college students are often considered a suitable population for research on executive functions due to their cognitive development and active engagement in academic activities. Secondly, college students typically have access to the necessary facilities and resources for participating in physical exercise programs, which makes it feasible to conduct this study within an academic setting. Lastly, college students are easily accessible for recruitment and are often willing to participate in research studies, which facilitates the recruitment process. The research hypotheses are as follows: (1) Higher-intensity physical exercise has a greater effect on executive function than longer durations. (2) A single session of physical exercise can enhance college students’ executive functions. (3) High-intensity physical exercise surpasses low-intensity physical exercise in sustaining the improvement of executive functions. This study investigates the effects of different exercise intensities and durations on executive functions in Chinese college students, while also exploring the potential interaction between these factors.

## 2. Materials and Methods

### 2.1. Participants

A mixed ANOVA design was employed. Initially, G*Power (3.1.9.7, Heinrich Heine University, Düsseldorf, Germany) was employed for power analysis during subject recruitment. According to the calculation method of the previous response size, 0.2 was a small effective size, and 0.5 was a medium effective size [26]. The selected variables were as follows: (1) Effect size f = 0.25; (2) α err rob of 0.05; (3) Power of 0.95; (4) Number of groups of 4; (5) Number of measurements of 4; (6) The expected attrition rate of 10%. It was calculated that at least 84 samples were needed to meet the research needs of this study.

**Figure 1:**
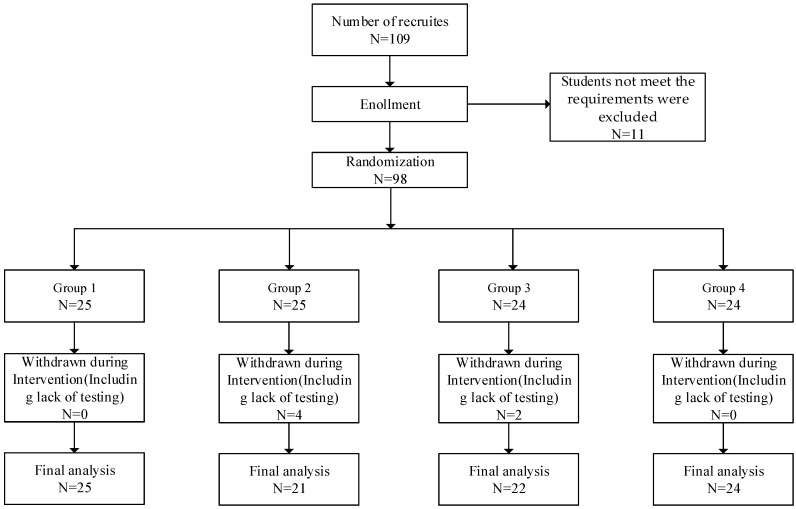
The flow diagram.

The study participants were recruited from Northeastern University, China. Undergraduate students were openly invited to participate in the experiment at Northeastern University in China. Undergraduate students who were physically and mentally healthy, with normal or corrected visual acuity, were recruited. The exclusion criteria for recruitment included: (1) experiencing a major traumatic event in the past year; (2) having a physical illness that hindered physical activity; (3) having an eye disease like color blindness or color weakness; and (4) having a severe mental illness, especially subjects on medication. Additionally, the inability to comply with the experimental rules was an additional exclusion criterion. After recruitment, a total of one hundred and nine college students enrolled in this study. After screening, eleven of them who did not meet the requirements were excluded. Ninety-eight subjects were divided into four groups to conduct the experiment. Ninety-eight subjects were randomly assigned to four groups using an online resource (http://www.randomization.com; accessed on 5 July 2023). Among the four assigned groups participating in the program, both male and female individuals were present. After conducting the experiments, six students dropped out because they missed the test or were unable to adhere to the experiments, and finally, ninety-two students met the requirements of the experiments to be included in the final analysis. Informed consent was obtained from all subjects, and this study was also approved by the Ethics Committee of Northeastern University (EC2022B025). The flow diagram was shown in Figure 1.

### 2.2. Procedure

The experiment spanned a duration of six weeks, during which training sessions were conducted twice a week. This study focused on assessing three core components of executive functions: working memory, inhibitory control, and cognitive flexibility. Prior to the commencement of training (time 1), a pre-test of executive functioning was administered to the college students. Subsequently, a second measure of executive functioning was conducted after the completion of the initial intervention (time 2). Following the final training session, a third measure of executive functioning was administered to the college students (time 3). Finally, six weeks after the conclusion of training (time 4), the participants underwent the final measure of executive functioning assessment.

### 2.3. Physical Exercise Methods

Recumbent bikes (STAR TRAC, Irvine, CA, USA) were used for physical exercise. The exercise intensity was classified into two categories: high-intensity and low intensity, while the exercise duration was categorized as either short or long. To monitor exercise intensity, the Borg RPE scale and the subjects’ heart rates were employed. The recumbent bike featured a real-time display of heart rate, enabling its use as a heart rate monitor. Exercise intensity is typically classified into three categories: low intensity, medium intensity, and high-intensity [27]. During the actual intervention, we found that it was difficult for the subjects to continue high-intensity exercise all the time, and after a period of high-intensity exercise, the subjects needed to take a short break or perform low-intensity exercises so their heart rate would decrease. Therefore, we included short breaks and low-intensity exercises during the test period in our analysis. In this study, we focused on two intensity levels, and therefore, we classified the parameters of moderate intensity exercise as high-intensity. Low-intensity was defined as a Borg RPE of 10–13 and less than 59% of the maximum heart rate (220-age). High-intensity was defined as a Borg RPE of 12–18 and 60–95% of the maximum heart rate. The workout duration was set at 20 min per session for the long duration category and 10 min per session for the short duration category. The subjects were divided into four groups based on the requirements of this study: Group 1: low intensity-short duration; Group 2: low intensity-long duration; Group 3: high-intensity-short duration; Group 4: high-intensity-long duration. Prior to the exercise, each group underwent a 5 min warm-up consisting of static stretching. In the event that subjects from the long-distance high-intensity group experienced difficulty sustaining the exercise, a 2 min break was permitted at the halfway point, after which the experiment resumed to ensure that the subjects’ heart rate remained at or above 60% of their maximum heart rate.

### 2.4. Measurement of Executive Functions

Working memory, inhibitory control, and cognitive flexibility in executive functions were tested as dependent variables.

Working memory: Working memory was assessed using the 2-back task developed by Kirchner [3]. The task involved a computer screen displaying nine spaces, with a square as the primary stimulus object. A stimulus object would appear in one of the nine spaces, and participants were required to determine whether its position matched the position of the stimulus presented two stimuli ago. They were instructed to press A if the positions matched or press L if they differed. Each stimulus was presented for 500 ms, with a 1500 ms interval between stimuli. A total of 60 test tasks were administered, preceded by 10 practice opportunities to ensure proficiency before the formal experiment commenced. Accuracy and reaction time were utilized as the primary indicators to evaluate working memory performance.

Inhibition control: Inhibition control was assessed using the Flank task developed by Eriksen and Eriksen [4]. Participants were presented with a row of 7 letters, consisting of the letters B and P. Their task was to identify the middle letter in the row. The order of the letters in each row was randomized, alternating between B and P. The letters were displayed for 1000 ms, and participants were required to respond within 2000 ms. Failure to respond within the allotted time was considered an error. Prior to the formal experiment, participants completed 10 pre-tests to familiarize themselves with the task. Once they became acquainted with the testing process, the formal experiment began. The entire experiment consisted of 60 trials. Accuracy and reaction time were selected as the indices for assessing inhibition control performance.

Cognitive flexibility: Cognitive flexibility was assessed using the More-odd shifting task developed by Derakshan [5]. The task involved presenting a series of numbers at the center of a computer screen. Each number was displayed for 2000 ms, with a stimulus interval of 1000 ms. Participants were instructed to make judgments regarding numbers 1–9, excluding 5. There were three types of judgments: (a) Odd and even judgments: When numbers were presented in red, participants had to determine whether they were odd or even. They were instructed to press A for odd numbers and L for even numbers. (b) Number size judgments: When numbers were presented in green, participants had to judge whether the number was greater than five or less than five. They were instructed to press A for numbers less than five and L for numbers greater than five. (c) Mixed judgments: When numbers were presented in red, participants performed odd and even judgments. When numbers were presented in green, they performed number-size judgments. The formal test consisted of six subsections, with a 20 s break between each subsection. Accuracy and reaction time in the mixed tasks were used as measures of cognitive flexibility and performance.

### 2.5. Statistical Analysis

Statistical analysis of the data was conducted using SPSS 26.0. All means and standard deviations were analyzed using standardized statistical methods. The normal distribution of the data was assessed using the Shapiro–Wilk test. A mixed ANOVA with a 2 (exercise intensity: low/high) × 2 (duration: short/long) × 4 (time of measurement: time 1/time 2/time 3/time 4) design was employed, with exercise intensity and exercise duration as independent variables and executive functions as the dependent variable. Sphericity tests were conducted using Mauchly’s test, and if sphericity assumptions were violated, the Greenhouse–Geisser correction was applied. The *p*-value was the level of significance, and a *p*-value value of less than 0.05 represents a significant difference. Bonferroni post hoc analyses were performed to examine significant differences. Eta squared was used to estimate effect sizes for significant main effects and interaction effects.

## 3. Results

### 3.1. Participant Characteristics

We used an exercise cardiorespiratory fitness testing system (Smax58ce, Highermed, Nanjing, China) to measure vital capacity and the International Physical Activity Questionnaire (IPAQ) to record the subject’s weekly physical activity. Participant characteristics were analyzed for gender (x^2^ (3) = 0.676), age (*F* (3, 88) = 0.973, *p* = 0.541), body mass index (*F* (3, 88) = 1.311, *p* = 0.205), vital capacity (*F* (3, 88) = 1.835, *p* = 0.119), resting heart rate (*F* (3, 88) = 1.912, *p* = 0.108), and physical activity (*F* (3, 88) = 1.930, *p* = 0.119). None of these characteristics showed significant differences (Table 1).

### 3.2. The Impact of Exercise Intensity and Duration on Executive Functioning

#### 3.2.1. Working Memory

**Figure 2:**
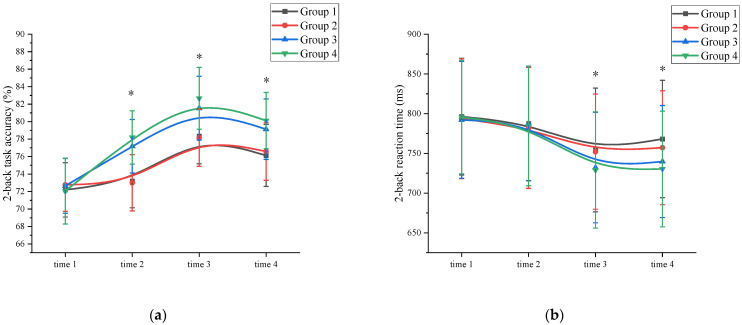
Working memory changes across the four measurements. *: asterisks indicate significant main effects at *p* < 0.05; (**a**): 2-back task accuracy; (**b**): 2-back task reaction time; Group 1: low intensity-short duration; Group 2: low intensity-long duration; Group 3: high intensity-short duration; Group 4: high intensity-long duration.

Accuracy: A mixed ANOVA analysis was conducted to examine the effects of exercise on working memory accuracy. The results revealed a significant main effect of measurement time on working memory performance (*F* (3, 89) = 79.876, *p* < 0.001, η^2^ = 0.319). Further analysis indicated that the accuracy of the time 3 measurement (80.18 ± 3.08) was significantly higher than that of time 1 (72.41 ± 3.18), time 2 (75.39 ± 3.33), and time 4 (77.99 ± 3.05) measurements (*p* < 0.05). Time 4 has a higher working memory accuracy than time 1. Additionally, a significant main effect of exercise intensity was observed (*F* (3, 86) = 117.112, *p* < 0.001, η^2^ = 0.574). Post hoc comparative analysis showed that at time 1, there was no significant difference (*p* > 0.05) between the high-intensity exercise groups (Group 3 (72.65 ± 3.14) and Group 4 (72.05 ± 3.77)) and the low-intensity exercise groups (Group 1 (72.19 ± 3.11) and Group 2 (72.77 ± 3.03)). At time 2, time 3, and time 4, the high-intensity exercise groups (Group 3 (time 2: 77.18 ± 3.06; time 3: 81.53 ± 3.65; time 4: 79.13 ± 3.46) and Group 4 (time 2: 78.19 ± 3.05; time 3: 82.67 ± 3.54; time 4: 80.12 ± 3.22)) were significantly different (*p* < 0.05) from the low intensity exercise groups (Group 1 (time 2: 73.19 ± 3.04; time 3: 78.34 ± 3.15; time 4: 76.13 ± 3.55)) and Group 2 (time 2: 73.00 ± 3.22; time 3: 78.16 ± 3.26; time 4: 76.58 ± 3.29)) (Supplementary Materials Table S1). The interaction between measurement time and exercise intensity was significant: *F* (3, 89) = 32.112, *p <* 0.001, η^2^ = 0.301. However, the main effect of exercise duration was not significant (*F* (3, 86) = 13.245, *p* = 0.105, η^2^ = 0.006). Further analysis of working memory accuracy at time 2 revealed that the high-intensity exercise group (77.69 ± 3.33) demonstrated a significant improvement (*p* < 0.05) compared to the time 1 measurements (72.35 ± 3.01). In contrast, the low-intensity exercise group showed improvement, but the difference was not statistically significant (*p* > 0.05). The interaction between measurement time and exercise duration was not significant (*F* (3, 89) = 11.231, *p* = 0.103, η² = 0.076). Demonstrated that exercise duration was not a major factor influencing working memory accuracy. These findings suggest that a single session of high-intensity exercise can effectively enhance working memory accuracy (Figure 2a and Supplementary Materials Table S1).

Reaction time: A mixed ANOVA analysis was conducted to examine the effects of exercise on reaction time in working memory. The results revealed a significant main effect of measurement time on working memory performance (*F* (3, 89) = 89.290, *p* < 0.001, η^2^ = 0.116). A comparison of reaction times showed that the time 3 measurement (741.96 ± 73.63) had significantly shorter reaction times compared to time 1 (794.48 ± 73.59), time 2 (785.50 ± 75.13), and time 4 (748.89 ± 74.46) measurements (*p* < 0.05). Time 4 has a shorter working memory reaction time than time 1. Additionally, a significant main effect of exercise intensity was observed (*F* (3, 86) = 89.128, *p* < 0.001, η^2^ = 0.305). Post hoc comparative analysis, at time 1 and time 2, there were no significant differences (*p* > 0.05) between the high-intensity exercise groups (Group 3 (time 1: 792.19 ± 73.59; time 2: 787.83 ± 72.11)) and Group 4 (time 1: 795.67 ± 71.48; time 2: 784.48 ± 75.32)) and the low-intensity exercise groups (Group 1 (time 1: 796.35 ± 73.49; time 2: 787.65 ± 72.18) and Group 2 (time 1: 793.69 ± 75.49; time 2: 782.05 ± 76.13)). At time 3 and time 4, the high-intensity exercise groups (Group 3 (time 3: 732.18 ± 69.48; time 4: 739.78 ± 70.50) and Group 4 (time 3: 729.15 ± 73.19; time 4: 730.42 ± 72.83)) were significantly different (*p* < 0.05) from the low-intensity exercise groups (Group 1 (time 3: 754.32 ± 77.82; time 4: 768.15 ± 73.91) and Group 2 (time 3: 752.17 ± 72.41; time 4: 757.19 ± 71.66)) (Supplementary Materials Table S2). The interaction between measurement time and exercise intensity was significant: *F* (3, 89) = 32.112, *p <* 0.001, η^2^ = 0.301. However, the main effect of exercise duration was not significant (*F* (3, 86) = 2.156, *p* = 0.117, η^2^ = 0.005). Further analysis indicated that the working memory reaction time at time 2 did not significantly differ from time 1 for all groups, suggesting that a single session of exercise was not sufficient to improve working memory reaction time. The interaction between measurement time and exercise duration was not significant (*F* (3, 89) = 14.572, *p =* 0.097, η^2^ = 0.113). Demonstrated that exercise duration was not a major factor influencing working memory reaction time. (Figure 2b and Supplementary Materials Table S2).

#### 3.2.2. Inhibition Control

**Figure 3:**
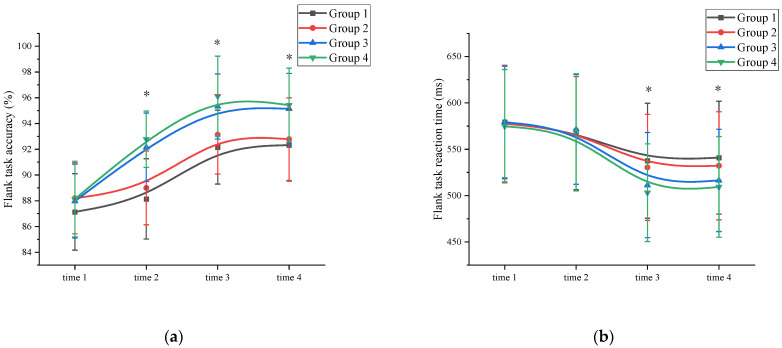
Inhibition control changes across the four measurements. *: asterisks indicate significant main effects at *p* < 0.05; (**a**): Flank task accuracy; (**b**): Flank task reaction time; Group 1: low intensity-short duration; Group 2: low intensity-long duration; Group 3: high intensity-short duration; Group 4: high intensity-long duration.

Accuracy: A mixed ANOVA analysis was conducted to examine the effects of exercise on inhibition control accuracy. The results revealed a significant main effect of measurement time on inhibition control performance (*F* (3, 89) = 84.592, *p* < 0.001, η^2^ = 0.215). Comparative analysis showed that the accuracy of the time 3 measurement (94.18 ± 2.97) was significantly higher than the accuracy of the time 1 (87.86 ± 3.05), time 2 (90.52 ± 3.13), and time 4 (93.91 ± 3.03) measurements (*p* < 0.05). Time 4 has a higher inhibition control accuracy than time 1. Additionally, a significant main effect of exercise intensity was observed (*F* (3, 86) = 125.467, *p* < 0.001, η^2^ = 0.328). Post hoc comparative analysis showed that at time 1, there was no significant difference (*p* > 0.05) between the high-intensity exercise groups (Group 3 (87.97 ± 2.87) and Group 4 (88.13 ± 2.94)) and the low-intensity exercise groups (Group 1 (87.13 ± 2.97) and Group 2 (88.19 ± 2.76)). At time 2, time 3, and time 4, the high-intensity exercise groups (Group 3 (time 2: 92.15 ± 2.66; time 3: 95.32 ± 2.53; time 4: 95.14 ± 2.75) and Group 4 (time 2: 92.78 ± 2.19; time 3: 96.12 ± 3.11; time 4: 95.43 ± 2.87)) were significantly different (*p* < 0.05) from the low intensity exercise groups (Group 1 (time 2: 88.14 ± 3.12; time 3: 92.16 ± 2.87; time 4: 92.32 ± 2.79)) and Group 2 (time 2: 89.00 ± 2.87; time 3: 93.13 ± 3.05; time 4: 92.78 ± 3.21)) (Supplementary Materials Table S3). The interaction between measurement time and exercise intensity was significant: *F* (3, 89) = 32.112, *p <* 0.001, η^2^ = 0.301. However, the main effect of exercise duration was not significant (*F* (3, 86) = 11.113, *p* = 0.069, η^2^ = 0.012). Further analysis of inhibition control accuracy at time 2 revealed that the high-intensity exercise group (92.47 ± 3.03) demonstrated a significant improvement (*p* < 0.05) compared to the time 1 measurements (88.05 ± 3.29). The interaction between measurement time and exercise duration was not significant; *F* (3, 89) = 7.124, *p =* 0.512, η^2^ = 0.031. Demonstrated that exercise duration was not a major factor influencing inhibition control accuracy. In contrast, the low-intensity exercise group showed improvement, but the difference was not statistically significant (*p* > 0.05). These findings suggest that a single session of high-intensity exercise can effectively enhance subjects’ inhibition control accuracy (Figure 3a and Supplementary Materials Table S3).

Reaction time: A mixed ANOVA analysis was conducted to examine the effects of exercise on reaction time in inhibition control. The results revealed a significant main effect of measurement time on inhibition control performance (*F* (3, 89) = 95.672, *p* < 0.001, η^2^ = 0.319). A comparison of reaction times showed that the time 3 measurement (520.63 ± 59.15) had significantly shorter reaction times compared to the time 1 (577.76 ± 61.34), time 2 (569.67 ± 63.98), and time 4 (524.69 ± 58.38) measurements (*p* < 0.05). Time 4 has a shorter inhibition control reaction time than time 1. The interaction between measurement time and exercise intensity was significant: *F* (3, 89) = 32.112, *p <* 0.001, η^2^ = 0.301. The main effect of exercise intensity was significant (*F* (3, 86) = 119.947, *p* < 0.001, η² = 0.412). Post hoc comparative analysis, at time 1 and time 2, there were no significant differences (*p* > 0.05) between the high-intensity exercise groups (Group 3 (time 1: 579.32 ± 60.25; time 2: 571.34 ± 59.14)) and Group 4 (time 1: 574.79 ± 61.23; time 2: 568.23 ± 63.25)) and the low-intensity exercise groups (Group 1 (time 1: 579.32 ± 61.23; time 2: 568.92 ± 62.57) and Group 2 (time 1: 577.59 ± 62.78; time 2: 570.18 ± 58.13)). At time 3 and time 4, the high-intensity exercise groups (Group 3 (time 3: 511.32 ± 56.73; time 4: 516.37 ± 55.15) and Group 4 (time 3: 503.14 ± 52.66; time 4: 509.32 ± 54.35)) were significantly different (*p* < 0.05) from the low-intensity exercise groups (Group 1 (time 3: 537.65 ± 61.99; time 4: 540.91 ± 60.87) and Group 2 (time 3: 530.42 ± 57.19; time 4: 532.17 ± 58.27)) (Supplementary Materials Table S4). The main effect of exercise duration was not significant (*F* (3, 86) = 2.156, *p* = 0.117, η^2^ = 0.005). Further analysis indicated that the inhibition control reaction time at time 2 did not significantly differ from time 1 for all groups, suggesting that a single session of exercise was not sufficient to improve inhibition control reaction time. The interaction between measurement time and exercise duration was not significant; *F* (3, 89) = 7.109, *p =* 0.205, η^2^ = 0.112. This demonstrated that exercise duration was not a major factor influencing the inhibition control reaction time (Figure 3b and Supplementary Materials Table S4).

#### 3.2.3. Cognitive Flexibility

**Figure 4:**
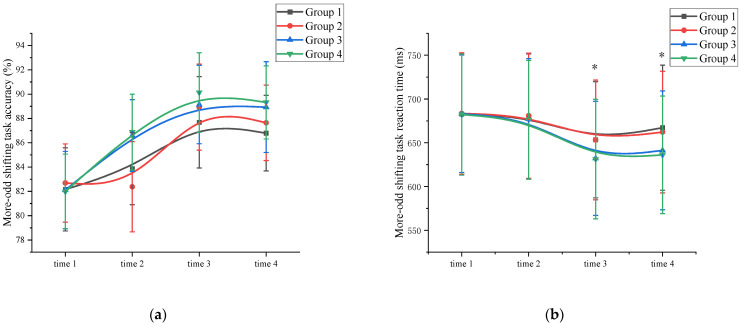
Cognitive flexibility changes across the four measurements. *: asterisks indicate significant main effects at *p* < 0.05; (**a**): More-odd shifting task accuracy; (**b**): More-odd shifting task reaction time; Group 1: low intensity-short duration; Group 2: low intensity-long duration; Group 3: high intensity-short duration; Group 4: high intensity-long duration.

Accuracy: A mixed ANOVA analysis was conducted to examine the effects of exercise on the accuracy of cognitive flexibility. The results revealed a significant main effect of measurement time on cognitive flexibility performance (*F* (3, 89) = 81.322, *p* < 0.001, η^2^ = 0.331). Comparative analysis showed that the accuracy of the time 3 measurement (88.97 ± 3.22) was higher than the accuracy of the time 1 (82.24 ± 3.11), time 2 (84.93 ± 3.25), and time 4 (88.17 ± 3.08) measurements (*p* < 0.05). Time 4 has a higher cognitive flexibility accuracy than time 1. Additionally, a significant main effect of exercise intensity was not observed (*F* (3, 86) = 66.263, *p* = 0.101, η² = 0.011). The interaction between measurement time and exercise intensity was significant, *F* (3, 89) = 32.112, *p <* 0.001, η^2^ = 0.301. However, the main effect of exercise duration was not significant (*F* (3, 86) = 17.321, *p* = 0.113, η^2^ = 0.027). Further analysis of cognitive flexibility accuracy at time 2 revealed that the high-intensity exercise group (86.74 ± 3.14) demonstrated a significant improvement (*p* < 0.05) compared to the time 1 measurements (82.05 ± 3.25). In contrast, the low-intensity exercise group showed improvement, but the difference was not statistically significant (*p* > 0.05). The interaction between measurement time and exercise duration was not significant, *F* (3, 89) = 13.111, *p =* 0.137, η^2^ = 0.059. This demonstrated that exercise duration was not a major factor influencing cognitive flexibility accuracy. These findings suggest that a single session of high-intensity exercises can effectively enhance subjects’ cognitive flexibility accuracy (Figure 4a and Supplementary Materials Table S5).

Reaction time: A mixed ANOVA analysis was conducted to examine the effects of exercise on the reaction time of cognitive flexibility. The results revealed a significant main effect of measurement time on cognitive flexibility performance (*F* (3, 89) = 82.167, *p* < 0.001, η^2^ = 0.109). Comparison showed that the reaction time measured at time 3 (642.52 ± 68.73) was significantly shorter than the reaction times measured at time 1 (682.88 ± 69.79), time 2 (678.53 ± 67.92), and time 4 (651.62 ± 68.13) (*p* < 0.05). Time 4 has a shorter cognitive flexibility reaction time than time 1. The interaction between measurement time and exercise intensity was significant, *F* (3, 89) = 32.112, *p <* 0.001, η^2^ = 0.301. However, the main effect of exercise intensity was significant (*F* (3, 86) = 112.369, *p* < 0.001, η^2^ = 0.412). Post hoc comparative analysis, at time 1 and time 2, there were no significant differences (*p* > 0.05) between the high-intensity exercise groups (Group 3 (time 1: 683.18 ± 67.15; time 2: 677.13 ± 68.93)) and Group 4 (time 1: 682.17 ± 69.14; time 2: 676.18 ± 67.85)) and the low-intensity exercise groups (Group 1 (time 1: 682.71 ± 68.65; time 2: 679.89 ± 71.58) and Group 2 (time 1: 683.46 ± 69.39; time 2: 680.93 ± 71.48)). At time 3 and time 4, the high-intensity exercise groups (Group 3 (time 3: 632.17 ± 65.14; time 4: 641.24 ± 67.88) and Group 4 (time 3: 631.27 ± 68.28; time 4: 636.16 ± 67.19)) were significantly different (*p* < 0.05) from the low-intensity exercise groups (Group 1 (time 3: 653.42 ± 66.39; time 4: 667.16 ± 71.40) and Group 2 (time 3: 653.22 ± 68.38; time 4: 662.19 ± 69.42)) (Supplementary Materials Table S6). The main effect of exercise duration was not significant (*F* (3, 86) = 3.156, *p* = 0.115, η^2^ = 0.009). Further analysis indicated that the cognitive flexibility reaction time at time 2 did not significantly differ from time 1 for all groups, indicating that a single session of exercise was not sufficient to improve cognitive flexibility reaction time. The interaction between measurement time and exercise duration was not significant (*F* (3, 89) = 9.408, *p =* 0.201, η^2^ = 0.105). This demonstrated that exercise duration was not a major factor influencing the cognitive flexibility reaction time. (Figure 4b and Supplementary Materials Table S6).

## 4. Discussion

This study demonstrates the effectiveness of physical exercise in enhancing the executive functions of college students in China. High-intensity groups prove to be more effective in improving executive functions compared to low-intensity groups. Moreover, there is not sufficient statistical evidence to show that longer-term exercise is more effective in executive functions. Additionally, this study examines the impact of a single exercise session on executive functions. The results indicate that a single session of high-intensity physical exercise yields superior improvements in executive functions among college students compared to low-intensity physical exercise. However, the effects of a single session of high-intensity physical exercise are limited to working memory accuracy, inhibitory control, and cognitive flexibility, without influencing reaction time. Conversely, a single session of low-intensity physical exercise does not affect executive functions. Importantly, the enhanced executive functions observed in the four groups persist at a certain level even after discontinuing physical exercise, highlighting the long-term effectiveness of exercise in maintaining executive functions. Two studies have demonstrated the positive effects of aerobic exercise on individuals’ executive functions [28,29]. Specifically, physical exercise has been found to effectively enhance working memory [30], inhibitory control [31], and cognitive flexibility [32]. This study further suggests that exercise intensity exerts a greater impact on improving executive functions compared to exercise duration.

This study provides evidence that physical exercise would enhance working memory in subjects. Specifically, a single session of high-intensity physical exercise improves the accuracy of working memory in subjects. However, it does not effectively improve the reaction time of working memory. On the other hand, prolonged physical activity proves to be effective in increasing both the accuracy and reaction time of subjects’ working memory. This study highlights the effectiveness of prolonged physical exercise in enhancing an individual’s thinking speed. Martins et al. demonstrated that moderate-intensity physical exercise significantly improves working memory [33]. In contrast, Kamijo and Abe conducted a physical intervention with middle-aged adults. Using bicycling, simple aerobic exercise appears to be more effective in improving working memory in this population [34]. Furthermore, Ludyga et al. demonstrated that the type of exercise also has a differential impact on an individual’s working memory [35]. Consistent with these findings, the present study reveals that exercise intensity exerts a greater effect on working memory compared to exercise duration. Exercise intensity is more effective in improving the working memory of the subjects.

Exercise intensity and duration have been shown to be effective in improving inhibitory control in college students. This study revealed that a single session of high-intensity physical exercise effectively enhances the accuracy of inhibitory control but does not impact the reaction time of the subjects. Following six weeks of exercise, both high-intensity and low-intensity exercise were found to improve the accuracy and reaction time of inhibitory control. Kao et al. demonstrated that a single 20 min session of aerobic exercise effectively improves individuals’ inhibitory control [36]. Consistent with these findings, our study shows that a single session of high-intensity physical exercise also enhances subjects’ inhibitory control. Nouchi et al. found that a single 30 min session of exercise improves inhibitory control in older adults [37]. However, prolonged exercise significantly enhances and sustains the level of inhibitory control in older adults over an extended period of time. Our study suggests that while a single session of high-intensity exercise effectively improves subjects’ inhibitory control, sustained exercise adherence leads to greater improvement and maintenance of inhibitory control over time.

Physical exercise has been shown to enhance cognitive flexibility in college students. This study found that a single session of high-intensity exercise significantly improved the accuracy of subjects’ cognitive flexibility. However, it did not have a significant impact on their reaction time. Following an extended period of training, both the accuracy and reaction time of cognitive flexibility were significantly improved in the subjects, and these improvements were sustained even after the completion of physical exercise. Netz et al. demonstrated that a single session of physical exercise significantly improved subjects’ cognitive flexibility [38]. Consistent with these findings, our study showed that a single session of intense physical exercise effectively enhanced the accuracy of subjects’ cognitive flexibility. Bae and Masaki elucidated the mechanism by which acute physical exercise improves cognitive flexibility using event-related potentials, highlighting that physical exercise induces changes in brain functioning, thereby affecting cognitive flexibility [39]. Our study further demonstrated that while a single session of intense physical exercise was effective in improving subjects’ cognitive flexibility, adherence to a prolonged period of exercise yielded greater improvements in cognitive flexibility.

This study demonstrated improvements in executive functions across all four groups. Specifically, high-intensity physical exercise was found to more effectively enhance the executive functions of college students. Furthermore, there is not sufficient statistical evidence to show that longer-term exercise is more effective in executive functions. Hsieh et al. emphasized the effectiveness of high-intensity interval training in improving executive functions [40]. This phenomenon can be attributed to the stimulation of the prefrontal cortex by high-intensity training, resulting in an immediate enhancement of executive functions. In our study, we also observed some improvement in executive functions with low-intensity physical activity. Yang highlighted the positive impact of low-intensity aerobic exercise on executive functions in older adults, making it a suitable exercise option for regular performance [41]. Regarding the influence of exercise duration on executive function improvement, our study indicated that exercise duration had a moderate effect, albeit smaller than exercise intensity. A single session of high-intensity exercise effectively improved executive functions, and the overall retention effect was superior to that of low-intensity exercise. Based on these findings, it can be concluded that high-intensity physical exercise is more effective in improving executive functions.

This study examines the impact of physical exercise on executive functions, with a specific focus on the effects of exercise intensity and duration on executive functions in college students. The findings demonstrate that high-intensity physical exercise yields superior improvements in executive functions compared to low-intensity physical exercise. Moreover, longer durations of physical exercise have a more pronounced effect on executive functions than shorter durations. Nonetheless, this study has some limitations. Firstly, this study lacks probability sampling methodology, and it solely employs a single form of physical exercise, specifically the recumbent bike, as the intervention. Secondly, this study primarily investigates the effects of exercise duration and intensity on executive functions, neglecting an examination of exercise frequency’s influence on executive functions. Thirdly, the study population included college students and did not involve other people (e.g., the elderly), so this study has some limitations.

## 5. Conclusions

Physical exercise has been shown to effectively enhance executive functions in Chinese college students. However, the impact of exercise intensity and duration on executive functions varies. High-intensity exercise has the potential to improve executive functions in college students within a single session. Furthermore, the benefits of high-intensity exercise extend beyond the immediate post-exercise period, leading to sustained improvements in executive functions. In terms of exercise duration, longer durations yield greater enhancements in college students’ executive functions compared to shorter durations.

## Figures and Tables

**Table 1:** Baseline characteristics of subjects (M ± SD).

Characteristics	Group 1	Group 2	Group 3	Group 4	*p*-Value
Number	25	21	22	24	—
Age(years)	20.86 ± 2.23	21.78 ± 2.96	22.16 ± 1.89	21.45 ± 2.11	0.541
Gender(males/females)	19/6	17/4	15/7	16/8	0.676
BMI (kg/m^2^)	22.31 ± 2.95	21.86 ± 3.07	22.06 ± 3.54	21.67 ± 2.88	0.205
Vital capacity (mL)	4003.46 ± 509.56	3908.37 ± 498.57	3947.38 ± 512.49	3912.34 ± 510.12	0.119
Resting heart rate	74.35 ± 8.13	74.57 ± 9.22	75.29 ± 8.55	76.56 ± 9.26	0.108
Physical activity (avg. min/day)	41.08 ± 10.34	47.67 ± 9.67	47.69 ± 10.29	43.48 ± 8.33	0.119

Abbreviations: M—mean; SD—standard deviation; BMI—body mass index.

## Data Availability

The data presented in this study are available on request from the corresponding author. The data are not publicly available due to privacy reasons.

## Supplementary Materials

The following supporting information can be downloaded at: https://www.mdpi.com/article/10.3390/bs13120987/s1, Table S1: A total of 2-back task accuracy (%); Table S2: A total of 2-back task reaction time (ms); Table S3: Flank task accuracy (%); Table S4: Flank task reaction time (ms); Table S5: More-odd shifting task accuracy (%); Table S6: More-odd shifting task reaction time (ms).
