# Supplementary material for: Effects of Physical Exercise on Executive Functions among College Students in China: Exploring the Influence of Exercise Intensity and Duration
Source: Behav Sci (Basel). 2023 Nov 29;13(12):987. doi: 10.3390/bs13120987 (PMC10740733; doi:10.3390/bs13120987)
Supplement: Supplementary file 1 [file behavsci-13-00987-s001.zip › behavsci-2660274-supplementary.pdf]

Table S1. A total of 2-back task accuracy (%).

	**Group 1**		**Group 2**		**Group 3**		**Group 4**		***p***
	M	SD	M	SD	M	SD	M	SD	
time1	72.19	3.11	72.77	3.03	72.65	3.14	72.05	3.77	0.312
time2	73.19	3.04	73.00	3.22	77.18	3.06	78.19	3.05	0.031
time3	78.34	3.15	78.16	3.26	81.53	3.65	82.67	3.54	0.025
time4	76.13	3.55	76.58	3.29	79.13	3.46	80.12	3.22	0.013
p	0.015		0.013		0.007		<0.001		

Note: M: mean; SD: standard deviations; *p*: *p*-value.

Table S2. A total of 2-back task reaction time (ms).

	**Group 1**		**Group 2**		**Group 3**		**Group 4**		***p***
	M	SD	M	SD	M	SD	M	SD	
time1	796.35	73.49	793.69	75.49	792.19	73.59	795.67	71.48	0.514
time2	787.65	72.18	782.05	76.13	787.83	72.11	784.48	75.32	0.103
time3	754.32	77.82	752.17	72.41	732.18	69.48	729.15	73.19	0.013
time4	768.15	73.91	757.19	71.66	739.78	70.5	730.42	72.83	0.016
p	<0.001		<0.001		<0.001		<0.001		

Note: M: mean; SD: standard deviations; *p*: *p*-value.

Table S3. Flank task accuracy (%).

	**Group 1**		**Group 2**		**Group 3**		**Group 4**		***p***
	M	SD	M	SD	M	SD	M	SD	
time1	87.13	2.97	88.19	2.76	87.97	2.87	88.13	2.94	0.425
time2	88.14	3.12	89.00	2.87	92.15	2.66	92.78	2.19	0.020
time3	92.16	2.87	93.13	3.05	95.32	2.53	96.12	3.11	0.025
time4	92.32	2.79	92.78	3.21	95.14	2.75	95.43	2.87	0.034
p	<0.001		<0.001		<0.001		<0.001		

Note: M: mean; SD: standard deviations; *p*: *p*-value.

Table S4. Flank task reaction time (ms).

	**Group 1**		**Group 2**		**Group 3**		**Group 4**		***p***
	M	SD	M	SD	M	SD	M	SD	
time1	579.32	61.23	577.59	62.78	579.32	60.25	574.79	61.23	0.317
time2	568.92	62.57	570.18	58.13	571.34	59.14	568.23	63.25	0.108
time3	537.65	61.99	530.42	57.19	511.32	56.73	503.14	52.66	0.003
time4	540.91	60.87	532.17	58.27	516.37	55.15	509.32	54.35	0.007
p	<0.001		<0.001		<0.001		<0.001		

Note: M: mean; SD: standard deviations; *p*: *p*-value.

Table S5. More-odd shifting task accuracy (%).

	**Group 1**		**Group 2**		**Group 3**		**Group 4**		***p***
	M	SD	M	SD	M	SD	M	SD	
time1	82.16	3.42	82.69	3.22	82.11	3.18	81.99	3.08	0.419
time2	83.87	2.97	82.38	3.71	86.59	2.95	86.88	3.12	0.078
time3	87.68	3.76	88.93	3.54	89.14	3.22	90.13	3.27	0.132
time4	86.79	3.11	87.64	3.1	88.93	3.73	89.32	3.01	0.113
p	0.012		0.005		0.004		<0.001		

Note: M: mean; SD: standard deviations; *p*: *p*-value.

Table S6. More-odd shifting task reaction time (ms).

	**Group 1**		**Group 2**		**Group 3**		**Group 4**		***p***
	M	SD	M	SD	M	SD	M	SD	
time1	682.71	68.65	683.46	69.39	683.18	67.15	682.17	69.14	0.421
time2	679.89	71.58	680.93	71.48	677.13	68.93	676.18	67.85	0.234
time3	653.42	66.39	653.22	68.38	632.17	65.14	631.27	68.28	0.018
time4	667.16	71.4	662.19	69.42	641.24	67.88	636.16	67.19	0.021
P	<0.001		<0.001		<0.001		<0.001		

Note: M: mean; SD: standard deviations; *p*: *p*-value.
